# Computational approaches towards the discovery and optimisation of cruzain inhibitors

**DOI:** 10.1590/0074-02760210385

**Published:** 2022-03-16

**Authors:** Viviane Corrêa Santos, Rafaela Salgado Ferreira

**Affiliations:** 1 Universidade Federal de Minas Gerais, Instituto de Ciências Biológicas, Departamento de Bioquímica e Imunologia, Laboratório de Modelagem Molecular e Planejamento de Fármacos, Belo Horizonte, MG, Brasil

**Keywords:** cruzain, cruzipain, Chagas disease, docking, molecular dynamics simulations

## Abstract

The need to develop safer and more efficacious drugs to treat Chagas disease has motivated the search for cruzain inhibitors. Cruzain is the recombinant, truncated version of cruzipain, a cysteine protease from *Trypanosoma cruzi* with important roles during the parasite life cycle. Several computational techniques have been applied to discover and optimise cruzain inhibitors, providing a molecular basis to guide this process. Here, we review some of the most recent computational studies that provided important information for the design of cruzain inhibitors. Moreover, we highlight the diversity of applications of *in silico* techniques and their impact.

One of the most employed approaches to find novel drugs to treat Chagas disease (CD) is the development of cruzain inhibitors. Cruzipains are a multigenic family of cysteine proteases from *Trypanosoma cruzi*, with varied roles during the parasite life cycle, associated with nutrition, metacyclogenesis, invasion of host cells, and modulation of macrophage response to infection.^1^ The term cruzain refers to the recombinant form of the most studied member of this family and is a truncated version at the C-terminal. Most of the medicinal chemistry literature, and all crystallographic complexes with inhibitors, have been determined with cruzain.

Cruzain’s active site is subdivided into seven sub-pockets able to accommodate the residues of the substrates. The pockets that accommodate the substrate amino acids (aas) from the acyl side are named S1 to S4, while the pockets accommodating the aas from the amino side are named S1’ to S3’. The catalytic triad Cys25, His162, and Asn182 locates in the interface between the S1 and S1’ pockets (Figure). Analysis of cruzain crystal structures reveals that the S3 to S1’ sub-pockets usually interact with inhibitors, being the most relevant for drug development.^2^ Among these pockets, only S2 is well defined, whilst the S1’, S1, and S3 pockets are shallow. Studies of cruzain’s substrate specificity indicate that S3 accommodates better positively charged residues and bulky aromatic groups; S2 accommodates hydrophobic aas, favoring the aromatic ones, and also accepting basic aas; S1 accommodates better positively charged residues and small aromatic groups; S1’ lacks an amino acid (aa) preference.^3^

**Figure:**
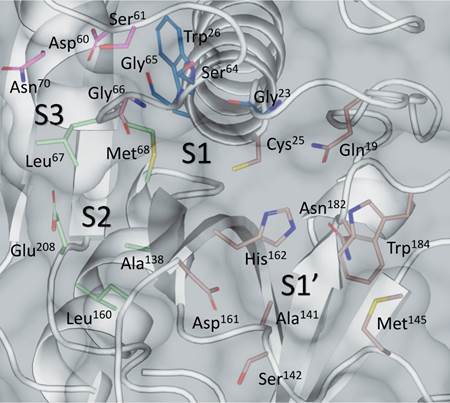
Cruzain’s active site residues and its division into sub-pockets. Residues from pockets involved in recognition of inhibitors are shown as sticks and colored according to the sub-pocket they belong to: pink (S3); green (S2); blue (S1); and brown (S1’). Cruzain structure (PDB 3KKU) is represented as cartoon and surface.

Throughout decades, several classes of cruzain inhibitors have been described (reviewed by da Silva et al.^1^ and Nascimento et al.^4^). Most times, computational techniques were employed to aid the drug discovery process. Here, we highlight key studies performed in the last four years. Even in this limited timeframe, we cannot provide a comprehensive summary in this perspective. Instead, we discuss a subset of studies that highlight the wide range of applications of computational techniques, exemplifying their power to aid the rational design of cruzain inhibitors (Table).

**TABLE:** Recent applications of computational techniques toward drug discovery targeting cruzain

Application	Computational techniques employed	Reference
Hit discovery	Docking-based virtual screening	^(5)^
Hit discovery and optimisation	Docking-based virtual screening	^(6)^
Ligand optimisation	Docking of analogs	^(7)^
	Construction of virtual libraries of analogs, docking-based virtual screening	^(8)^
Binding mode prediction	Docking	^(9)^
	Docking, molecular dynamics simulations, MM-GB/SA calculations	^(10)^
	Docking	^(13)^
Prediction of binding affinity	Thermodynamic integration	^(11)^
Understanding SAR	Molecular dynamics simulations, analysis of contact profiles	^(11)^
	Molecular dynamics simulations, machine learning	^(12)^
	Thermodynamic fingerprints from molecular dynamics simulations	^(13)^
Understanding mechanism of inhibition	QM/MM simulations	^(14)^
Generation of models for affinity prediction	QSAR models	^(15)^
Predicting allosteric sites	Molecular dynamics simulations, docking-based virtual screening, and MM-GB/SA	^(16)^
Description of cruzipain sub-types	Genomic analysis, phylogenetics, transcriptomic analysis, comparative modeling	^(2)^

MM-GB/SA: molecular mechanics-generalised born/surface area; SAR: structure-activity relationships; QM/MM: quantum mechanics/molecular mechanics; QSAR: quantitative structure-activity relationship.

Structure-based discovery and optimisation of cruzain inhibitors

A common strategy to search for drug candidates is to test millions of molecules against the target evaluating the biological outcomes; in the case of cruzain, researchers look for inhibitors. Experimental screening of a massive quantity of molecules is time-consuming, expensive, and requires intensive laboratory work in confirmatory assays. Thus, virtual screening techniques have been used to accelerate the hit discovery process and diminish the costs in the last decades. Virtual screening hits are molecules predicted to be active against the target, and they must be assayed to confirm the predictions. One method widely applied in virtual screening campaigns is molecular docking. It predicts the binding mode between two molecules, the biological target and the small molecule under investigation. These calculations require the 3D structural data from the target and the molecules. To date, 28 crystal structures of cruzain complexed with small molecules, and one apo structure, have been experimentally determined. This type of information had been used in docking-based virtual screening campaigns. Silva et al*.* ^5^ screened a library of 120 small natural and nature-based compounds with docking and selected 14 naphthoquinone-based molecules for the enzymatic assays. They found three cruzain inhibitors, one with a potency in the low micromolar range (IC_50_ 6.3 µM). De Souza et al.^6^ used three different docking algorithms to screen almost 4,000,000 molecules from the lead-like and fragment-like subsets of the ZINC database. The ZINC database contains over 750 million purchasable compounds for virtual screening and is broadly used in the community. The compounds are divided into subsets organised according to the molecules’ molecular properties. Based on this screening, the authors selected 14 lead-like and four fragment-like compounds for the assays. They found one cruzain inhibitor between the fragment-like molecules, with an IC_50_ of 1.0 µM (Compound **1**). The subsequent design of analogs for compound **1** yielded optimised inhibitors, with an IC_50_ of 120 nM for compound **45**. The superposition of the predicted binding mode of both compounds suggests a conservative binding mode between them. In another study, Ferreira et al.^7^ used molecular docking to predict the binding mode between cruzain and compound **3a** (IC_50_ of 2.2 µM), a previously reported competitive inhibitor. The compound hydrogen bonds with Gly66, Asp161, and Gln19, as commonly observed in crystallographic complexes of cruzain bound to inhibitors. Based on these results, they synthesised analogs that were tested against cruzain, resulting in a nanomolar range inhibitor **10j** (IC_50_ of 0.6 µM). The predicted binding mode for **10j** maintains the hydrogen bond pattern of **3a**, conserving the same binding mode. Once hits are known, docking-based virtual screening can also aid in prioritising analogs for synthesis. Starting with a micromolar-range cruzain inhibitor (compound **1a**, IC_50_ of 7.5µM), Barbosa da Silva et al.^8^ screened 3,365 analogs and selected 22 for synthesis and biological assays, resulting in an optimised compound **1s** with an IC_50_ of 2.5 µM.

Molecular docking studies can also be very useful in proposing the binding mode of known cruzain competitive inhibitors, helping to generate hypotheses about the importance of different compound moieties for binding. Pereira et al.^9^ have predicted binding modes of two competitive non-covalent cruzain inhibitors, and one of them was later optimised through a Structure-Based Drug Discovery (SBDD) approach.^8^ More sophisticated pipelines have also been described toward ligand binding mode prediction. Martins et al.^10^ combined docking with molecular dynamics (MD) simulations and molecular mechanics-generalised Born/surface area (MM-GB/SA) calculations to compare possible binding modes of a quinoline derivative. Considering evidence from multiple techniques, they selected one of the major MD ligand clusters as the most likely binding mode of the inhibitor, based on its higher stability in simulations, lower internal ligand strain, and predicted binding affinity.

Understanding structure-activity relationships (SAR) and mechanism of inhibition

SAR studies frequent follows the description of an inhibitor series. Knowing the SAR for a compound series can be useful in ligand optimisation, especially if rationalised in terms of protein-ligand binding interactions. Santos et al.^11^ performed MD simulations and thermodynamic integration (TI) calculations to understand the SAR of a benzimidazole series of cruzain inhibitors. TI recapitulated relative binding affinity with high accuracy, while the interactions observed in the simulations indicate an important role of the bromophenyl ring and the linker region, bound at the S2 subsite, to anchor the compounds. More specifically, they frequently observed hydrogen bonds to Gly66 and Asp161, besides van der Waals interactions with several residues in the S2 pocket.^11^ In another study, Luchi et al.^12^ combined quantum theory, MD simulations, and machine learning to analyse a series of 17 vinyl sulfone cruzain inhibitors, divided into two groups according to their binding affinity. Their analysis indicates that potent inhibitors induce conformation changes in S3, S2, and S1’ subsites, favoring positioning of the warhead and cruzain conformations expected for substrate recognition. Compared to the less potent compounds, the most actives interact more frequently with Gly66, Asp161, Gly163, and Leu67. Lameiro et al.^13^ studied the binding of peptidomimetic nitriles by covalent docking and MD. By simulating three inhibitors, they investigated two SAR trends observed for the series: the impact of stereochemistry and a substitution in P2. As reported by other studies, the authors highlight the importance of hydrogen bonds to Gly66 and Asp161 and hydrophobic interactions to Leu67 for affinity to cruzain.

Computational studies have also provided insight for understanding differences in the mechanism of cruzain binding to different classes of covalent inhibitors. To do so, Silva et al.^14^ studied a pair of inhibitors that differed only in the warhead, representing two classic classes of cruzain inhibitors: vinyl sulfones and nitriles. Quantum Mechanics/Molecular Mechanics (QM/MM) and MD simulations were used to explore the reaction mechanism between cruzain and the two compounds. In agreement with experimental data, the ΔG of the reaction with the vinyl sulfone was much more negative, as expected for an irreversible inhibitor. The study also indicated a difference in the reaction mechanism: while the Cys25 nucleophilic attack and His162 proton transfer occur in a single step for a reversible inhibitor, these events occur in two steps for an irreversible covalent inhibitor.^14^

Another computational technique applied to understand SAR trends is the quantitative structure-activity relationship (QSAR), a ligand-based approach in which models are built to correlate mathematically the chemical features of molecules with their biological activity. These models are built based on sets of molecules with activity described against the target of interest. QSAR can predict whether a molecule will be active or to comprehend which modifications improve the activity in a compound series. Thus, QSAR models have been used in virtual screening for hit identification and ligand optimisation steps. For example, Rosas-Jimenez et al.^15^ searched in the ChEMBL database for molecules with reported IC_50_ against cruzain. With a 344 inhibitors dataset, they built predictive QSAR predictive models to find chemical diverse cruzain inhibitors. These models are available in a Python script.

Getting biological insights relevant for drug discovery

Most of the studies to find cruzain inhibitors search for molecules able to bind to the orthosteric site. However, cruzain belongs to the papain family of cysteine proteases, sharing high structural similarity with the other family members, such as human cathepsins L and B, leading to selectivity problems. Thus, developing inhibitors able to bind to allosteric sites is a way to overcome these challenges. In that sense, Álvarez et al.^16^ employed computational structural techniques to identify and characterise a putative allosteric site in cruzain. They started mapping the human cathepsin K (hCatK) allosteric sites in the cruzain structure and, aided by MD simulations, identified two novel allosteric sites with at least 200 Å^3^ volume calculated with POVME (POcket Volume Measurer). These sites were validated with a docking-based virtual screening, MD simulations, and MM-GB/SA. The authors also analysed the cruzain residues interacting with the ligands and compared them with the hCatK ones to discuss a possible selectivity mechanism.

Another challenge in working with cruzain as a drug target is that it belongs to the cruzipain gene family. Despite most of the drug studies focusing on cruzain, there are other cruzipain sub-types expressed by *T. cruzi*. Santos et al.^2^ identified two cruzipain families organised in two clusters in the parasite genome and subdivided the sequences into four sub-types. Family I embraces the czp1 sub-type, the target of drug design projects, represented by cruzain. Family II embraces the czp2, czp3, and czp4 sub-types. These divisions were made based on alterations observed on the cruzipains’ active sites and are predicted to affect the interaction of the enzymes with their substrates and small molecules. The authors modeled the tertiary structure of Family II members using a cruzain structure as a template and compared the residues in the active site of each cruzipain sub-type. They also analysed RNAseq data and described that Family I sequences are expressed through all the parasite life stages. However, this expression is more pronounced in the epimastigote forms. On the other hand, Family II sequences are more expressed in the trypomastigotes, which, together with amastigotes, are more relevant in the drug design context.^2^ These findings raise a red flag to groups working with cruzain as a drug target. Researchers have known the existence of over one cruzipain for a long time. However, it has been neglected by the community. This work suggests the need to understand the role and specificity of the four sub-types and potentially consider all of them in the drug discovery pipeline targeting cruzipains. Alterations observed in different cruzipain subsites might cause selectivity between them and could explain inconsistencies between enzymatic and cell-based assays reported in the literature. On the other hand, there are enough similarities in the sites that can also be explored to design inhibitors active against all the four sub-types.

Final remarks

Cruzain is an important target in developing new treatments for CD. In this perspective, we exemplify several applications of computational techniques toward this goal. In recent years, computational approaches have been critical to discovering and optimising new classes of cruzain inhibitors, proposing binding modes of known compounds, and understanding SAR trends from compounds series in qualitative and quantitative terms. Additionally, we have learned about the biology of this target with the proposal of allosteric sites and the identification of several cruzipain sub-types. Altogether, these studies demonstrate the potential of computational techniques to optimise drug discovery efforts, potentially reducing the time and costs involved in this process. These benefits are particularly welcome in efforts to develop drugs for neglected diseases.
